# A qualitative study of hospital pharmacists and antibiotic governance: negotiating interprofessional responsibilities, expertise and resource constraints

**DOI:** 10.1186/s12913-016-1290-0

**Published:** 2016-02-06

**Authors:** Alex Broom, Stefanie Plage, Jennifer Broom, Emma Kirby, Jon Adams

**Affiliations:** 1 School of Social Sciences, The University of New South Wales, Sydney, NSW 2052 Australia; 2 Department of Medicine, Sunshine Coast Hospital and Health Service, Nambour, QLD 4560 Australia; 3 University of Technology, Sydney, NSW 2007 Australia

**Keywords:** Antibiotics, Qualitative research, Semi-structured interviews, Pharmacy, Hospital governance, Antimicrobial resistance, Australia, Antimicrobial Stewardship, Antibiotic prescribing

## Abstract

**Background:**

Antibiotic treatment options for common infections are diminishing due to the proliferation of antimicrobial resistance (AMR). The impact of Antimicrobial Stewardship (AMS) programs seeking to preserve viable antibiotic drugs by governing their use in hospitals has hitherto been limited. Pharmacists have been delegated a critical role in antibiotic governance in AMS teams within hospitals but the experience of pharmacists in influencing antibiotic use has received limited attention. In this study we explore the experiences of pharmacists in antibiotic decision-making in two Australian hospitals.

**Methods:**

We conducted 19 semi-structured interviews to explore hospital-based pharmacists’ perceptions and experiences of antibiotic use and governance. The analysis was conducted with NVivo10 software, utilising the framework approach.

**Results:**

Three major themes emerged in the pharmacist interviews including (1) the *responsibilities of* pharmacy in optimising antibiotic use and the interprofessional challenges therein; (2) the importance of antibiotic streamlining and the *constraints* placed on pharmacists in achieving this; and (3) the potential, but often under-utilised *expertise,* pharmacists bring to antibiotic optimisation.

**Conclusions:**

Pharmacists have a critical role in AMS teams but their capacity to enact change is limited by entrenched interprofessional dynamics. Identifying how hospital pharmacy’s antibiotic gatekeeping is embedded in the interprofessional nature of clinical decision-making and limited by organisational environment has important implications for the implementation of hospital policies seeking to streamline antibiotic use. Resource constraints (i.e. time limitation and task prioritisation) in particular limit the capacity of pharmacists to overcome the interprofessional barriers through development of stronger collaborative relationships. The results of this study suggest that to enact change in antibiotic use in hospitals, pharmacists must be supported in their negotiations with doctors, have increased presence on hospital wards, and must be given opportunities to pass on specialist knowledge within multidisciplinary clinical teams.

**Electronic supplementary material:**

The online version of this article (doi:10.1186/s12913-016-1290-0) contains supplementary material, which is available to authorized users.

## Background

Antimicrobial resistance (AMR) is a prominent public health concern. While the proliferation of multi-drug resistant pathogens reaches pandemic proportions, the development of new antibiotic drugs has stagnated, presenting the significant potential for a post-antibiotic era in the near future [1–4]. The repercussions for global health are immense, yet one of the drivers of the acceleration of antibiotic resistance remains elusive to change—the excessive and inappropriate use of antibiotics in health service settings. Optimising antibiotic use in healthcare settings, in addition to curbing use in agriculture, is a key domain for safeguarding antibiotics for future generations [2, 5]. In particular, studies document widespread suboptimal prescribing in hospitals in Europe, North America, Australasia and many OECD countries [6–8]. Research in Australian hospitals, for example, shows that while 50 % of all inpatients receive antibiotics [9], between 20–50 % of assessable prescriptions are inappropriate [10].

### The limited impact of antimicrobial stewardship (AMS)

In the past decade a range of clinical governance programs aiming at optimising antimicrobial use in hospitals have been developed under the umbrella term of *antimicrobial stewardship* (AMS) [11–16]. These initiatives have focused largely on influencing doctors’ behaviour given that they are the prescribers [17]. Evidence indicates, however, that AMS programs are not achieving sustained changes in practice either in Australia or internationally [6, 8–10] and there is considerable uncertainty as to how to improve clinical outcomes [18]. While there is research emerging regarding the complex factors that influence doctors’ antibiotic decisions [17], including institutional norms limiting practice change [19], there has been little examination of the experiences of non-physician stakeholders [20, 21]. As shown in the results presented below, hospital work is interprofessional in character and effective antibiotic governance requires a multidisciplinary approach [11, 12, 14, 22].

### The role of pharmacy in the hospital and antibiotic governance

Traditionally the pharmacist’s role was the dispensing of drugs at the request of the doctor [23]. In the last few decades, pharmacy has expanded beyond dispensing medications to being part of clinical teams on the hospital wards and assuming management roles [23, 24]. Pharmacy is an increasingly important stakeholder at the bedside alongside nursing and medicine shifting professional focus to *pharmaceutical care* and individual patients’ drug therapy [25–27]. More recently, pharmacists began to assume additional responsibilities in the governance of antibiotics including controlling the availability of drugs in the hospital [11], education and raising awareness about AMR among nursing and medical staff [22], and providing audit and feedback regarding antibiotic use [10, 14, 22]. However, despite these shifts it is unclear how and in what ways pharmacists are influencing antibiotic prescribing, directly and indirectly.^1^ The aim of this study is to begin to fill this gap in knowledge by examining the experiences of hospital pharmacists in influencing antibiotic use and governance. In particular, we document themes in their views on their ability to promote concordance with therapeutic guidelines, their relationships with doctors, and any constraints to their participation in prescribing decisions.

## Methods

### Design

This is a qualitative study utilising semi-structured interviews with Australian hospital-based pharmacists involved in antibiotic decision-making.

### Participants and sampling

Once ethics approval was granted by The University of Queensland and The Prince Charles Hospital Human Research Ethics Committees (HREC #2013000029) the study was advertised to all pharmacists working at the two participating hospitals. Both hospitals are acute regional, small to mid-size institutions with approximately 500 beds between them. They are affiliated with a university-based clinical school providing training to up to 20 medical students at any given time. As is required by the Australian Commission for Safety and Quality in Healthcare National Standards, the participating hospitals have an active antibiotic stewardship program in place including a post-prescription approval system for restricted antimicrobials, with an integral role for pharmacy in identifying restricted antimicrobial prescriptions. A multidisciplinary AMS committee with representation from medical, nursing, pharmacy and executive staff meets monthly, and is chaired by an Infectious Diseases Physician. An AMS pharmacist participates in ward rounds twice weekly reviewing antimicrobial prescriptions. Audit and feedback is a critical component of the AMS programme, involving multiple specialty areas.

Over 90 % (*n* = 29) of the contacted pharmacists agreed to participate. The researchers (AB and EK) visited the hospitals on a series of scheduled days in mid-2014, and completed interviews with all available participant volunteers. Before the interviews, participants received an information sheet about the study and had the opportunity to ask questions prior to signing the consent form. Nineteen of the pharmacists were available during the scheduled fieldwork days. The researchers agreed following completion and analysis of the 19 interviews that data saturation had been reached, i.e. new data did not offer any additional insights into the questions posed by this study [28]. The sample included pharmacists of three career ‘Health Practitioner’ levels; HP3 requires a pharmacy degree (*n* = 9), HP4 requires a pharmacy degree and a post-graduate qualification (*n* = 7), and HP5/HP6 are management position or advanced-level practitioners (*n* = 3). Pharmacists also self-reported as being “early-career” (*n* = 6), “mid-career” (*n* = 6), or “senior” (*n* = 7) in the interviews. There was as a gender split (12 females and 7 males) reflective of the hospitals’ pharmacist population.

### Interview protocol

The interviews utilised a thematic guide derived from the relevant literature and flexibly incorporated topics raised by the participants as the interviews progressed (see Additional file 1). Such approach allowed both systematic data collection and ongoing development of analytical categories sensitive to the emergent themes in the data [28, 29]. The interviews were broadly structured around the following areas: pharmacists’ views on antibiotic use and resistance; the role of pharmacists within the hospital regarding antibiotics; professional and interprofessional issues; and, governance and organisational dynamics. For the purpose of this study, we defined ‘optimal prescribing’ as the appropriate choice of antibiotic, route of delivery and duration of antibiotic therapy [9]. We used prompts and probes to invite participants to reflect on antibiotic use and governance in their hospital department. All interviews lasted between 30 and 60 min, were audio recorded and fully transcribed. We de-identified all references to hospital locations, clinics’ names or individuals in the data to preserve anonymity.

### Data analysis

During our data analysis we continually sought to retain the richness of the respondents’ experiences, documenting atypical cases, conflicts, and contradictions within the data [30]. Upon identifying a theme we searched through all the interviews for other related comments, employing constant comparison to develop or complicate them. This process helped ensure that events initially viewed as unrelated could be grouped together as their interconnectedness became apparent. The final step involved revisiting the literature and seeking out conceptual tools that could be employed to make sense of the patterns that had emerged from the data [30]. NVivo10 software was used to support our thematic content analysis using the framework approach allowing comparisons within and across cases while maintaining the analytical complexity of the participants’ narratives [28, 31, 32].

## Results

Three major themes emerged around the perceptions of hospital pharmacy’s *responsibilities* in the streamlining of antibiotics use, the *constraints* within which these activities are performed and the *expertise* pharmacists potentially bring to the bedside.

### Professional responsibilities of pharmacy within the hospital and antibiotic governance

All participants displayed a strong sense of professional identity and outlined their perceptions with respect to prescribing in both practical and general terms:

*“I: What is the purpose of pharmacy?*

*P: It is the appropriate use of drugs, the right drug for the right indication for the right patient, and the right dose”* (Senior, HP4).

Another participant commented on what hospital pharmacy *does:*

*“… tackling issues when they come up; annotating your charts appropriately; contacting doctors when [prescribing]’s deemed inappropriate; asking for advice from people when you’re not sure; taking the time to look things up if you don’t know the answer yourself. And it’s ingrained in us through our practice from the very beginning, this is what you’re taught […], because that's basically what a pharmacist does is they’re a problem solver”* (Senior, HP3).

All respondents agreed that pharmacy’s main function is to be the clinical team’s experts on medications driven by their professional education, identity and standards. This translates into communication with doctors, giving and seeking advice, keeping records and establishing links across different departments of the hospital. In everyday practice, pharmacists assume a range of different responsibilities when solving problems around antibiotic prescribing:

*… as pharmacists we don’t make choices other than to intervene. And our role is predominately advisory”* (Senior, HP4).

*“… a hands-on approach to them, to keep on advising and recommending and so on. Everyone has access to the antibiotic guidelines, but it’s getting them [doctors] to do it”* (Senior, HP4).

*“… It’s pretty obvious that they [doctors] aren’t aware of the restrictions to prescribing certain antibiotics and it’s up to pharmacy to make them aware”* (Mid-career, HP5/HP6).

Pharmacists interpreted their role in antibiotic prescribing within the boundaries of their relationship with doctors—i.e. they acted as doctors’ advisors, provided education and got involved more directly in antibiotic decision-making. Hospital pharmacy’s potential to bring drug expertise to bear is associated with its greater penetration into the provision of care at the bedside. Increasingly, pharmacy assumes duties beyond the dispensing of drugs, requiring stronger involvement in clinical practice and a physical presence on hospital wards. As this senior pharmacist described, ideally:

*“[pharmacists] spend their day … in the wards rather than living out of a pharmacy department. … the idea is that they’re readily available to all the doctors at any time. … So we do act as a team, so we try and do a little bit of everything. So at any one time one of us could be doing anything for anyone on any ward”* (Senior, HP4).

*“It’s clear if you have experienced pharmacists as part of the team I could guarantee that would improve antimicrobial prescribing. And that's not just for serious things like septicaemia, that's from everything…trained pharmacists make a huge impact, there's just not enough, and we’re always behind the eight ball”* (Senior, HP3).

The interviews revealed environmental and resource constraints limiting pharmacists’ ability to ensure their clinical presence and contribute proactively to antibiotic decisions. This perception was voiced across all levels of seniority and areas of clinical focus. Pharmacists made sense of these constraints and their implications for everyday work in different ways:

*“There's a basic delivery of services which needs to happen and that is the delivery of medication, and everything else is extra. So if you can’t, you have to ensure that the core service is functional … until you can cover your core work … and we’re doing it well, I don’t see how we can then say ’well we’ll take on the responsibility of ensuring that we do antibiotic education, and we’re rolling out guidelines, and we’re making sure we’re picking up suboptimal prescribing and we’re looking at gentamicin lists and we’re doing all the stuff that we can do to capture this problem'”* (Senior, HP4).

*“… So it’s an enormous amount of work… there's a job where you clock in and you clock out. And I think if you’re going to be a professional maybe there's a perception … it needs to become your vocation as well. So that thing that you do out of hours for enjoyment maybe that needs to be cut down a bit because you’re going to need to put some hours in at home”* (Senior, HP3).

It emerged that pharmacy’s ongoing clinical expansion remains somewhat incomplete. The participants expressed an inability to work towards consistent antibiotic practices in accordance with hospital policies during working hours. They acknowledged new responsibilities within the tighter governance of antibiotic use, but described a process of prioritisation as a result of competing demands on their time. This prioritisation revealed that a “basic delivery of services” (i.e. the generic dispensing of drugs) was perceived as core business with responsibilities around promoting the judicious use of antibiotics being described as “extras” by pharmacists on the wards. There are clear role-based limitations on the penetration of pharmacy expertise at the bedside and in the capacity of pharmacy to help streamline antibiotic use.

### Informal constraints on pharmacy’s antibiotic gatekeeping practices

These constraints are made more pertinent by the informal practices pharmacists face when attempting to optimise antibiotic use in line with antibiotic guidelines. Almost all of our participants talked about difficulties to intervene once a prescription was written. Being present at the moment of initiating antibiotic therapy was for most the exception. This, and the lack of a default mechanism to correct suboptimal antibiotic scripts, exposed pharmacy interventions to a range of inconsistencies in practice. We asked pharmacists how they deal with prescriptions that deviate from therapeutic guidelines:

*“We cannot supply it … we all carry the list of ID approval requirements. They’re not in the impres[t] rooms [onsite drug supply], so then it does have to go through pharmacy. And then pharmacy can say 'hey we’re not going to do this'. So that's our biggest bargaining chip”* (Early career, HP3).

The imprest stock is an onsite supply of medications accessible by nurses and doctors at any time. All antibiotic drugs that need approval from an Infectious Diseases specialist are stocked only in limited quantity. For a supply of more than 24 h specific dispensing from pharmacy is required for each patient. This gives pharmacy the ability to track the use of restricted drugs. Many of the pharmacists emphasised the importance of this measure of control and regarded themselves as “gatekeepers” watching over the use of antibiotic drugs:

*“… we seem to be the people that are sort of the gatekeepers … and we seem to always be the ones going 'well no you can’t have that. You have to get ID approval'”* (Mid-career, HP4).

*“… we have to be the gatekeeper a bit in the middle of the nurses and the doctors. Yeah we’re very responsible for it, if we end up with none [antibiotics] left to choose from in the future, if we just gave everything out willy nilly, that's the reason I guess why everything’s not on imprest, or everything’s not in the after hours cupboard, because there's meant to be a pharmacist involved in that”* (Early career, HP3).

*“… We can then go and say ‘okay we’ve now got thirty patients, we’ll look at charts to see how inappropriate prescribing is. Let’s see the areas or the teams or who are prescribing inappropriately.’ We then need to feedback to them to say ‘look there's a problem here. These are actually the guidelines, this is what we need to do. Would you like some assistance in writing a protocol? Do you need education?’”* (Senior, HP4).

Managing and restricting the supply of antibiotic drugs is one of the key antimicrobial stewardship interventions of hospital pharmacy. Pharmacists justified this enactment of interprofessional control emphasising their responsibility to safeguard the viability of antibiotics for the future. The latter interview excerpt above demonstrates how restrictive management of supply is accompanied by attempts to educate and raise awareness about therapeutic guidelines. Participants also discussed the limitations a restrictive approach to streamlining antibiotic use imposes. The same participant as above admitted that:

*“… pharmacy can certainly be a platform because they have the ability to restrict the actual supply. And that's not necessarily the right answer…”* (Senior, HP4)

*“… there isn’t enough time obviously for pharmacy to both review it and supply it. So it’s hard. You’re talking about a system that has to still function. How much do you restrict it? How much can you restrict it?”* (Early career, HP3).

The participants doubted that gatekeeping practices alone were the best way to optimise antibiotic use. The latter interviewee above viewed it even as potentially harmful to “the functioning of the system”. This argument relates to the hospital as an organisational setting characterised by hierarchically-structured professional relations. Establishing a dispensing authority in the form of the pharmacist as gatekeeper alongside the prescribing authority manifest in the doctor was perceived as a threat to the integrity of that system. The majority of participants voiced concerns about the appropriateness and effectiveness of this part of their work:

*“So it depends on the ward, whether it’s on the impres[t], like in their drug rooms. Then the nurses just have the straight out availability to go and take it. … If a pharmacist doesn’t see it it can just go under the radar. … also, if they don’t have it on their ward they will go to another, the respiratory ward that does have it and get it just to avoid pharmacy”* (Early career, HP3).

*“… they’ll just go to a different ward, or they’ll change it to something easier that someone's not going to hassle them about, but might not be the best choice. I think that's a big problem is a lot of the time they will do something easier, that doesn’t require ID [Infectious Diseases approval] … they’ll choose a different antibiotic rather than choosing the correct one and just giving them a call”* (Early career, HP3).

Restrictive supply management on its own was described by the above, and other participants as ultimately counterproductive and encouraging suboptimal choice of antibiotic drug rather than providing decision aids for problematic cases. These reported informal practices undermined these pharmacists’ capacity to enforce best practice guidelines and put pressure on them to engage directly with doctors in discussions about appropriate antibiotic prescribing. These negotiations take place between distinct professional communities drawing on different types of knowledge resources, cultural idiosyncrasies and sets of responsibilities.

### Pharmacy’s antibiotic expertise at the bedside

Gatekeeping practices alone are ineffective and may have adverse effects on the choice of antibiotics pushing pharmacy to intervene more directly in the prescribing process. Participants’ accounts of their involvement expanded beyond the choice of drug into common prescribing issues around duration, dosage and mode of delivery. While some pharmacists dismissed doctors as “ignorant” or even “lazy”, others explained suboptimal prescribing practices in terms of different knowledge resources, professional standards, localised norms and differing notions of care, i.e. pharmaceutical care as narrowly focused on individual drug therapy and clinical care as taking a holistic approach to patient assessment and treatment. More precisely they perceived it as a clash between medical judgment and pharmaceutical standards. The following participants discussed how they view pharmacy’s drug expertise fitting into clinical practice:

*“… for us [pharmacy] our main focus all the time is drugs. And I'm aware that for the doctors, they’ve got so many other things that they have to think about. And so sometimes for them they’ve just got to get whatever it is written down and look at everything else”* (Mid-career, HP4).

*“… We’re pharmacy based so we’re thinking about drugs all the time … Whereas they [doctors]’ve got a much broader spectrum. … we’re pretty privileged as being pharmacists in hospitals that we get to look at the holistic view of the patient, we are able to look at their labs, we can see their sensitivities, … we’ve had four-and-a-half … years training looking at medications and seeing if they’re appropriate. And then we can relay that to the doctors”* (Early career, HP3).

The pharmacists understood their expertise as unique and highly specialised on drugs as opposed to the broader remit of medical knowledge. They framed this expertise as different from, and more specialised than, the clinical knowledge of the medical profession. Most participants agreed that the value of pharmacy is not to supplant medical judgment but provide practical advice and prompts to complement it, for example, by assisting with precise dosing, raising awareness about AMR and promoting the accessibility of clinical guidelines. Pharmacists built on their professional education and standards to argue for their potential to make meaningful contributions in the clinical field. They situated their argument within an ethical discourse about the “patient good” which served as an overarching framework connecting pharmacy with the medical profession. This subtheme became more evident when they talked about the frustrations they experience when their drug expertise clashed with prescribing decisions made by doctors:

*“… if [as a doctor] you've seen something prescribed over ten years and you know it’s going to work you know, it might be 90 % right. … You can go up to a doctor and go ‘why did you choose this’ or whatever, and it’s like’close enough is good enough’, … As long as they’ve got something that's treating the patient and they’re going to get better, then I don’t think they care too much”* (Mid-career, HP4).

*“Well close enough is not good enough! … ‘I think the dose is okay, it won’t kill them, it’ll do’, that's terrible, that is absolutely terrible. … we’re in an electronic age, it’s at your fingertips, it’s not that hard to look up. … I would no sooner prescribe something that I wasn't completely sure what the dose was, if I could prescribe, than fly to the moon! You know that's terrible! It’s a patient!”* (Mid-career, HP4).

*“… you see it all the time and you go’that's not great but it’s safe, it’s a little bit effective’ and … you've got to pick your battles … we’re still often giving supply because it’s either the patient gets no antibiotics, or a patient gets something that - you know we can sort of look from our end I guess and say ’yeah they’re covering the right bug, it’s probably not the best therapy’”* (Early career, HP3).

There was a high degree of inconsistency within the interviews regarding how the participants resolved the tension between their drug expertise and allowing for medical judgment and thus reducing conflict. Individual pharmacists make choices to what extent they follow-up cases of suboptimal prescribing. While some argued prescribing is either done right or wrong and deviations from best practice were challenged, others engaged in “picking battles” and perceived antibiotic prescribing as a grey zone. The first group of participants drew on collective quality standards within the pharmacy profession to enforce precise dosing, appropriate choice of drug, duration and mode of delivery. They supported their argumentation by pointing out the instant availability of knowledge resources online and off-line, laboratory testing with short turnarounds and pharmacy and ID counsel. Ultimately, they utilised discourses about the “patient good” to justify demands on the medical profession for greater accuracy in antibiotic prescribing. The second group engaged in a process of prioritisation and de-escalation guided by pragmatic principles taking into account resource constraints of time-poor doctors, the multitude of clinical factors doctors have to consider beyond drugs and the apparent—albeit limited—efficacy of the course of antibiotic treatment. They also drew on similarly understood discourses about the “patient good” to defend poor prescribing as long as patient outcomes were positive. Both groups placed short-term patient outcomes rather than long-term concerns about AMR at the centre of their ethical reasoning moderating the moral distress of sub-optimal antibiotic practices [33]. In either group ideas about the “patient good” were used to bridge the professional gap between pharmaceutical and medical care and implicitly confirm pharmacy’s legitimate presence at the bedside.

## Discussion

The proliferation of AMR has resulted in attempts to better govern the use of antibiotics in hospitals. However, recent changes to antibiotic governance with a focus on altering doctors’ behaviour have had limited success. Our previous research revealed the significance of other stakeholders in antibiotic decision-making [17], and a variety of AMS approaches acknowledge, in particular pharmacists’ potential to make a meaningful contribution to streamlining antibiotics use [11, 12, 14, 20]. This study reveals three important insights into hospital-based pharmacists’ experiences of antibiotic prescribing.

First, this study indicates that hospital pharmacists perceive themselves as antibiotic gatekeepers seeking to fulfil educational, advisory and supervisory roles within antibiotic governance—they negotiate with other stakeholders caring for patients at the bedside to bring their expertise on drugs to bear and are empowered by their control over the supply of antibiotics. This reaffirms the interprofessional nature of antibiotic decision-making and the need to address potential institutional and informal barriers to enhanced collaboration across professionals and settings in the hospital.

Second, we found that some doctors and nurses can undermine pharmacists’ dispensing of restricted antibiotic drugs, thus weakening their ability to prevent deviations from therapeutic guidelines and offer timely advice on drug choice, mode of delivery and duration. This highlights the potential detrimental effects of interprofessional dynamics and resource constraints on hospital pharmacy‘s capacity to perform responsibilities pertaining to antibiotic governance. Increased presence on the hospital wards and better integration into clinical teams would provide more opportunities to pharmacists to get involved in antibiotic decision-making, ideally prior to prescriptions being written.

Third, consistent with previous research on the reporting of prescribing errors [34], pharmacists in our sample dealt inconsistently with suboptimal antibiotic prescriptions brought to their attention. The individual pharmacists reported that they made choices about when to intervene and seek to correct or optimise antibiotic treatment decisions. Some stressed the importance of concordance with therapeutic guidelines and pharmaceutical best practice whereas others strategically challenged inaccuracies in antibiotic prescribing, and selectively so.

However, this study is not without limitations. Given the qualitative approach of the study, the findings within this context cannot be generalized to other settings. The AMS program implemented at the participating hospitals favours, besides educational measures and evaluation, the restrictive management of antimicrobials through its antibiotic approval system in which pharmacy is tasked with gatekeeping duties. Pharmacists working in settings with no antibiotic restrictions, or with AMS programs which rely on other mechanisms may encounter different issues. Our data indicated that there exists a tension between clinical judgment and pharmaceutical expertise which requires significant exploration. Only if the reasoning behind pharmacists’ decisions to prioritise and intervene in the optimisation of antibiotics use and its relation to medical expertise is understood, institutional mechanisms can be designed to promote more consistent engagement of hospital pharmacy in antibiotic governance.

## Conclusions

Our findings have significant repercussions for antibiotic optimisation and streamlining. The increasing self-perception of pharmacy as antibiotic gatekeeper opens up significant potential for individual pharmacists to influence antibiotic decision-making in everyday practice. However, the resistance of doctors to pharmacy advice and the inconsistencies in pharmacy practice impose severe constraints on the implementation of hospital policies aiming at the optimisation of antibiotic prescribing. These need to be addressed, for example via the design of institutional mechanisms that are sensitive to the differences in professional culture and provide pharmacy with a stronger foothold at the bedside. This includes the reallocation of resources to acknowledge hospital pharmacy’s responsibilities beyond the dispensing of medicines and provide them with opportunities to accumulate specialised clinical experience. Increased presence of pharmacists on hospital wards across high resource and low resource settings would also allow the establishment and maintenance of trusting relationships between pharmacists and doctors that promotes a sense of shared obligation to the “patient good” and a mutual understanding as partners in the effort to curb the proliferation of AMR.

## Additional file

Additional file 1:
**Interview Schedule and Indicative Interview Questions.** (DOCX 15 kb)

## Abbreviations

AMR: antimicrobial resistance
AMS: antimicrobial stewardship

## Footnotes

1 In Australia, for example, the automatic stop order system allows them to effectively nullify antibiotic treatment decisions made by doctors if these are in violation of institutional procedures, such as lacking timely approval of restricted drugs by an Infectious Diseases specialist. Little is known however, to what extent pharmacists exercise this measure of control in practice.
