# Supplementary material for: A qualitative study of hospital pharmacists and antibiotic governance: negotiating interprofessional responsibilities, expertise and resource constraints
Source: BMC Health Serv Res. 2016 Feb 6;16:43. doi: 10.1186/s12913-016-1290-0 (PMC4744423; doi:10.1186/s12913-016-1290-0)
Supplement: Additional file 1: — Interview Schedule and Indicative Interview Questions. (DOCX 15 kb) [file 12913_2016_1290_MOESM1_ESM.docx]

**Additional file 1: Interview Schedule and Indicative Interview Questions**

*1: Perceptions of the wider debate*

- To what extent are you aware of debates around resistance/antibiotic prescribing?
- What do you think are the most significant contributors to the development of antibiotic resistance locally and globally?
- Who do you perceive to be responsible and who has the capacity to address this issue?

*2: Perspectives on the role of pharmacy within the hospital*

- Could you please talk a little bit about the typical circumstances in which you are involved in the use and delivery of antibiotics?
- What is the role of pharmacy in decisions about antibiotics and the use of antibiotics in the hospital?
- To what extent does pharmacy engage in, or input into, decisions about antibiotic use in the hospital?
- What clinical situations are the most challenging from a pharmacy perspective regarding antibiotics?

*3: Knowledge and expertise*

- How knowledgeable do you feel, in your day-to-day clinical work, regarding antibiotic choices?
- What education/training would be helpful regarding antibiotic use from a pharmacy perspective?
- Can you explain how you would help to establish ‘best practice’ in antibiotic prescribing?

*4: Inter-professional issues*

- Could you please talk about your interactions with other professional groups within the hospital within the context of antibiotic use (e.g. doctors and nurses)?
- How would you describe your relationship with the Infectious Diseases department, and to what extent does this improve/problematise your everyday delivery of antibiotics?
- What happens in contexts of inter-professional disagreements (e.g. between specialties/clinician groups) and how (and to what extent) are these resolved across illness contexts?
- What formal or informal processes take place in decision-making about antibiotic use according to seniority/experience?

*5: Governance*

- What is your knowledge of the audit/governance processes around antibiotic prescribing and to what extent are these reasonable/appropriate?
- To what extent are local governance procedures working?
